# Determination of Optimal Fluoroscopic Angulations for Left Main Coronary Artery Ostial Interventions: 3-Dimensional Computed Tomography Validation

**DOI:** 10.1155/2022/2411824

**Published:** 2022-03-10

**Authors:** Yuhe Sheng, Jie Yu, Quanmin Jing, Yaling Han, Yi Li, Kai Xu, Miaohan Qiu, Yingdong Wang, Gary S. Mintz, Bin Wang

**Affiliations:** ^1^Department of Cardiology, General Hospital of Northern Theater Command, Shenyang, China; ^2^Cardiovascular Research Foundation, NY, NY, USA

## Abstract

**Background:**

Current recommendations for the best views for the left main coronary artery (LMCA) ostium intervention are empirical.

**Objectives:**

To determine the optimal projection to visualize the LMCA ostium using only fluoroscopy.

**Methods:**

The optimal projection to visualize the LMCA ostium was determined using fluoroscopic images of superimposing the lowest points of the distal ends of two *J* tipped wires in the noncoronary cusp (NCC) and right coronary cusp (RCC). This was validated independently using 3-dimensional computed tomography (3D-CT) reconstruction.

**Results:**

Satisfactory images of the overlapping wires in NCC and RCC could be obtained in 90% (45/50). Between the fluoroscopic and the 3D-CT reconstruction approaches, the mean difference for NCC and RCC overlapping at horizontal axes is -1.8 with a 95% limit of agreement between −3.94 and 0.34 (*p*=0.10) and at vertical axes −1.6 with a 95% limit of agreement between −3.46 and 0.26 (*p*=0.09); and the mean difference for the optimal projection to visualize the LMCA ostium at horizontal axes is −3.22 with a 95% limit of agreement between -7.26 and 0.81 (*p*=0.11) and at vertical axes −2.31 with a 95% limit of agreement between −5.83 and 1.21 (*p*=0.09). The 3D angulation deviation for the optimal projection to visualize the LMCA ostium was 8.5° ± 4.7° when the LMCA ostium faced the NCC-RCC commissure (*n* = 32) and 22.3° ± 16.0° (*p*=0.009) when it did not (*n* = 13).

**Conclusions:**

The optimal projection for LMCA ostial intervention can be determined using fluoroscopic images of superimposing wires in the NCC and RCC when the LMCA ostium faces the NCC-RCC commissure, as was the case in 71% of the patients studied.

## 1. Introduction

A good fluoroscopic working view provides essential anatomical landmarks that are particularly crucial during left main coronary artery (LMCA) ostial stent implantation, and the stent must cover the LMCA ostium without too much protrusion to the aorto that would make future intervention more difficult [1, 2]. On one hand, current recommendations for the most appropriate fluoroscopic working views are empirical [3]. On the other hand, the optimal fluoroscopic working view can be determined using 3-dimensional computed tomography (3D-CT) reconstruction software if the patient has undergone coronary CT or electrocardiographically gated ascending aortic CT before the angiography-guided procedure [4, 5]. It has not been reported if the optimal fluoroscopic working view for LMCA ostial stent implantation can be determined using only fluoroscopic images. We propose an approach based on fluoroscopic images of overlapping wires in the aortic cusps and validate this approach with 3D-CT reconstruction software.

## 2. Materials and Methods

### 2.1. Patient Population and Study Design

The study included 50 consecutive patients scheduled to undergo coronary angiography after coronary CT assessment from April 2021 to June 2021 in our center. All patients provided written informed consent. The optimal projection to visualize the LMCA ostium was generated by superimposing wires in the aortic cusps. The optimal projection to visualize the LMCA ostium was also generated by a 3D-CT reconstruction software independent of angiography. The difference between the optimal projection by the two approaches would be evaluated finally.

### 2.2. Determination of the Optimal Projection to Visualize the LMCA Ostium Using Fluoroscopic Images

**Figure 1:**
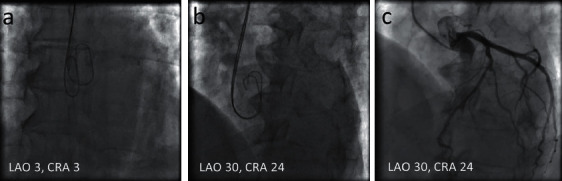
Optimal projection to visualize the LMCA ostium determined by fluoroscopic images of the wires. LAO = left anterior oblique; CRA = cranial.

The optimal projection to visualize the LMCA ostium was estimated using fluoroscopic images of superimposing wires in the noncoronary cusp (NCC) and right coronary cusp (RCC). The radial artery or femoral artery was used for access. Two *J* tipped 0.035-inch wires were advanced to the NCC and RCC of the aortic valve using the anterior-posterior view (Figure 1(a)). Usually, the first wire would be advanced into the NCC. The second wire could be advanced into the RCC after being tried several times. The hydrophilic soft wire could be considered for use if the second wire did not work. If the second wire could not be advanced to the RCC at the anterior-posterior view, the C-arm could be moved to the right anterior oblique (RAO) and caudal. In this view, the wire in the NCC would be at the left side and the wire in the RCC would be at the right side. Then, the C-arm was rotated in the left anterior oblique (LAO) and cranial directions until the lowest points in the curve at the distal part of these two wires were superimposed (Figure 1(b)), in which NCC and RCC would be considered as overlapping. This view angulation of the overlapping NCC and RCC was considered as the optimal projection to visualize the LMCA ostium. The LMCA ostial image was recorded using angiography of the left coronary artery (Figure 1(c)).

### 2.3. Determination and Validation of the Optimal Projection to Visualize the LMCA Ostium by CT Software

**Figure 2:**
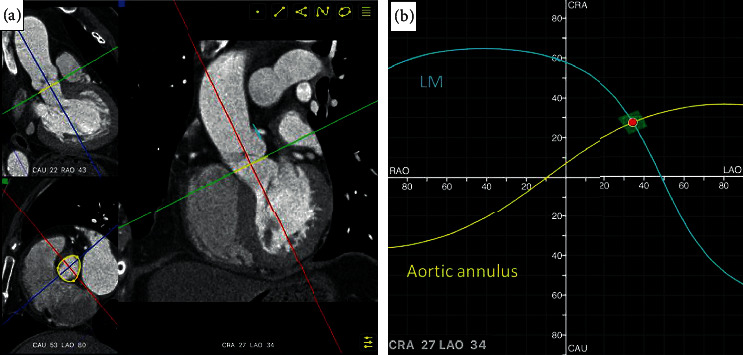
Optimal projection to visualize the LMCA ostium estimated by FluoroCT: (a) the aortic annulus (yellow line) and LMCA plane (blue line) were determined; and (b) the optimal projection of the LMCA ostium was fixed when two S-curves consisting of an unlimited number of pairs of C-arm angulations which were tangential to the aortic annulus and the LMCA ostium crossed. LAO = left anterior oblique; RAO = right anterior oblique; CRA = cranial; CAU = caudal.

Validation of the optimal projection to visualize the LMCA ostium independent of angiography was performed by 2 independent physicians using 3D-CT reconstruction software (FluoroCT software version 3.0 developed by Pascal Thériault-Lauzier and Nicolo Piazza). The CT images of the 75% time phase were imported into FluoroCT. The reference lines were placed at the middle of the coronary and sagittal planes in the aortic root. The aortic annulus was determined when the nadirs of three coronary cusps were seen simultaneously by moving the transverse plane vertically. The LMCA ostium plane could also be fixed in the same way (Figure 2(a)). The two S-curves consisting of an unlimited number of pairs of C-arm angulations that were tangential to the aortic annulus and LMCA ostium would cross. Angulation of the crossing point would be the optimal projection to visualize the LMCA ostium from FluoroCT (Figure 2(b)) [5].

**Figure 3:**
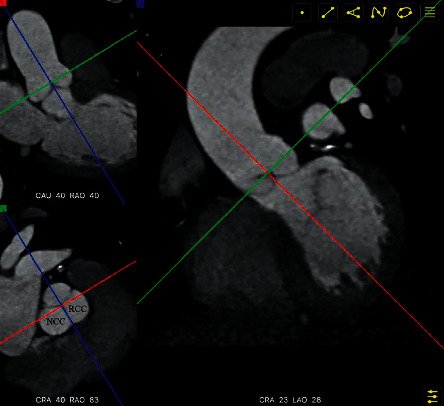
The blue reference line crossed the center of the cusp plane and the NCC-RCC commissure (bottom left corner). The NCC and RCC overlapped when viewed in the coronary plane. The angulation of NCC and RCC overlapping would be seen at the bottom of the coronary plane (right). NCC = noncoronary cusp; RCC = right coronary cusp. LAO = left anterior oblique; RAO = right anterior oblique; CRA = cranial; CAU = caudal.

The transverse plane at the aortic annulus was moved up to the plane where the three cusps were seen clearly. The reference lines on the transverse plane were rotated until one crossed the center of the cusp plane and the NCC-RCC commissure. The NCC and RCC would overlap on the coronary plane. Concurrently, the angulation of the NCC and RCC overlap would be determined (Figure 3).

**Figure 4:**
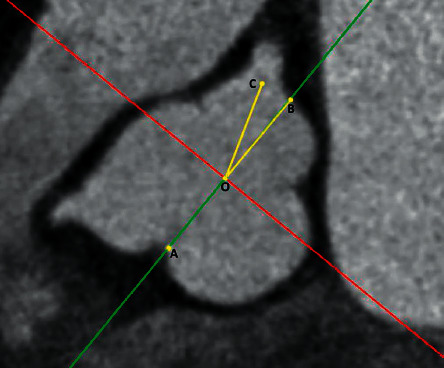
Definitions of the angle between the LMCA ostium and the NCC-RCC commissure. ‘Dot A' was at the NCC-RCC commissure. ‘Dot O' was at the center of the cusp plane of the aortic valve. ‘Dot B' was at the opposite side of dot A on the cusp plane. ‘Dot C' was at the center of the LMCA ostium. The angle between the LMCA ostium and NCC-RCC commissure was ∠BOC. The LMCA ostium was considered to be facing the NCC-RCC commissure when ∠BOC was equal to 0°.

The angle between the LM ostium and the NCC-RCC commissure was measured. The center of the cusp plane was defined as the angular vertex. The line crossing the commissure and the center of the cusp plane was defined as one side of the angle. The line crossing the center of LMCA ostium and the center of the cusp plane was defined as another side of the angle. The ostium was considered to be facing the NCC-RCC commissure when the LMCA ostium was on the line crossing the commissure and the center of the cusp plane (Figure 4).

Using *α* to denote the C-arm LAO/RAO angle and *ß* for the cranial/caudal angle, the C-arm position was denoted as (*α*, *β*). The first C-arm position (*α*1, *β*1) defined a direction in space (unit vector) which we denoted as (x1, y1, z1), where *x*1 = Sin (*α*1) Cos (*β*1), *y*1 = Sin (*β*1), and *z*1 = Cos (*α*1) Cos (*β*1). The second C-arm position (*α*2, *β*2) was described with (x2, y2, z2), where *x*2 = Sin (*α*2) Cos (*β*2), *y*2 = Sin (*β*2), and *z*2 = Cos (*α*2) Cos (*β*2). The 3D deviation of the two C-arm positions (*δ*), calculated in degrees, was given by the formula *δ* = Cos^−1^(x1x2 + *y*1y2 + *z*1z2) [6, 7].

### 2.4. Statistical Analysis

Variables were presented as the mean ± standard deviation or, if the distribution was not Gaussian, as the median and interquartile range. A Bland–Altman analysis was conducted to compare the angle measurements in fluoroscopy versus CT. Independent samples were compared using the 2-tailed unpaired Student's *t*-test. Analyses were performed using IBM SPSS 22.0 software. Statistical significance was set at *p* < 0.05 (2-sided).

## 3. Results

In 90% of the patients (45/50), it was possible to advance the wires to the NCC and RCC and obtain satisfactory overlapping images of the two wires. In the 45 patients, the age of the patients was 61 [54–66]; 76% (34/45) of the patients were male; the height of the patients was 168 ± 7 centimeters; the weight was 72 [65–83] kilograms; and the body mass index was 25.6 [24.5–27.9] kg/m^2^. The 0.035-inch wires could not be advanced to NCC and RCC in two patients, and satisfactory overlapping images of the two wires could not be obtained in three patients.

### 3.1. Comparison of the NCC and RCC Overlapping between Fluoroscopy and CT

**Figure 5:**
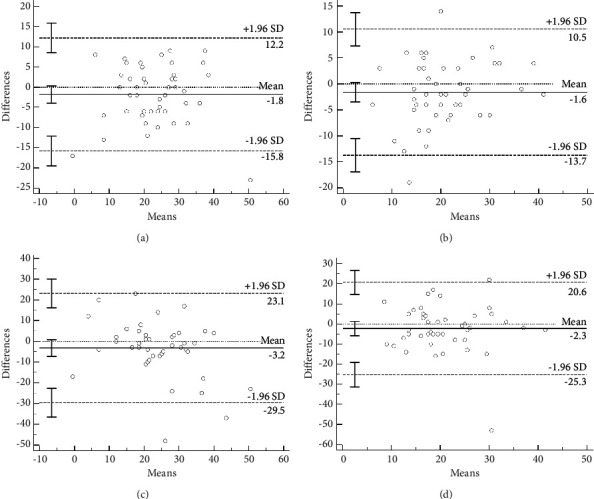
Comparison of the NCC and RCC overlapping between coordinates generated using each method on (a) horizontal and (b) vertical axes and comparison of the optimal projection to visualize the LMCA ostium between coordinates generated by each method on (c) horizontal and (d) vertical axes.

The angles of the overlapping NCC and RCC wires as seen using fluoroscopy were compared with the angles of overlapping NCC and RCC as seen using FluoroCT. The 3D deviation between these two methods was 8.0° ± 4.4°. The mean fluoroscopy-derived angle of the overlapping NCC and RCC was 22.2 ± 10.1, while the mean FluoroCT-derived angle of the overlapping NCC and RCC was 24.0 ± 10.1 at LAO. The mean fluoroscopy-derived angle of the overlapping NCC and RCC was 19.8 ± 8.9, while the mean FluoroCT-derived angle of the overlapping NCC and RCC was 21.4 ± 7.6 at cranial. The Bland–Altman analysis showed a consistency between these two methods. The mean difference for the angle of the overlapping NCC and RCC at the horizontal axes was −1.8 with a 95% limit of agreement between -3.94 and 0.34 (*p*=0.10). The mean difference for the angle of the overlapping NCC and RCC at vertical axes was −1.6 with a 95% limit of agreement between −3.46 and 0.26 (*p*=0.09) (Figure 5(a) and 5(b)).

### 3.2. Comparison of the Optimal Projection to Visualize the LMCA Ostium between Fluoroscopy and CT

The 3D deviation of optimal projection to visualize the LMCA ostium between these two methods was 12.5° ± 11.2°. The mean fluoroscopy-derived the optimal projection to visualize the LMCA ostium was 22.2 ± 10.1, while the mean FluoroCT-derived angle of the overlapping NCC and RCC was 25.4 ± 14.2 at LAO. The mean fluoroscopy-derived angle of the overlapping NCC and RCC was 19.8 ± 8.9, while the mean FluoroCT-derived angle of the overlapping NCC and RCC was 22.1 ± 9.8 at cranial. The Bland–Altman analysis showed a consistency between these two methods. The mean difference for the optimal projection to visualize the LMCA ostium at horizontal axes was −3.22 with a 95% limit of agreement between −7.26 and 0.81 (*p*=0.11). The mean difference for the optimal projection to visualize the LMCA ostium at vertical axes was −2.31 with a 95% limit of agreement between −5.83 and 1.21 (*p*=0.19) (Figures 5(c) and 5(d)).

### 3.3. Comparison of 3D Angulation Deviation of the NCC-RCC Overlapping between the LMCA Facing the NCC-RCC Commissure or Not

**Figure 6:**
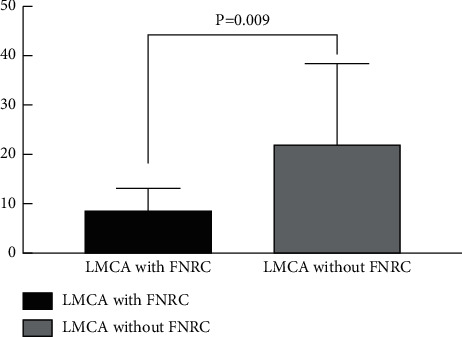
Comparison of the 3D deviation in the optimal projection to visualize the LMCA ostium between fluoroscopy and CT comparing patients in whom the LMCA ostium faced the NCC-RCC commissure (*n* = 32) vs. patients in whom the LMCA ostium did not face the NCC-RCC commissure (*n* = 13). LMCA = left main coronary artery; FNRC = face to the NCC-RCC commissure.

Overall, in 71% (32/45) of the cases, the LMCA ostium faced the NCC-RCC commissure. The 3D angulation deviation between the NCC and RCC overlapping and the optimal projection to visualize the LMCA ostium from FluoroCT was 3.1° ± 2.9° in patients in whom the LMCA ostium faced the NCC-RCC commissure. Twenty percent (9/45) of the LMCA ostium faced the RCC, while 9% (4/45) of the LMCA ostium faced the NCC. The 3D angulation deviation of the optimal projection to visualize the LMCA ostium between fluoroscopy and CT determinations was 8.5° ± 4.7° in patients in whom the LMCA ostium faced the NCC-RCC commissure versus 22.3°+ ± 16.0° in patients in whom the LMCA ostium did not face the NCC-RCC commissure (*p* = 0.009) (Figure 6). The supplemental appendix displayed all the data and graphs showing the comparison of the NCC and RCC overlapping and the optimal projection to visualize the LMCA ostium between fluoroscopy and CT when the LMCA ostium faced the NCC-RCC commissure or not.

### 3.4. Examples of LMCA Ostial Intervention by the Approach

#### 3.4.1. Example One

**Figure 7:**
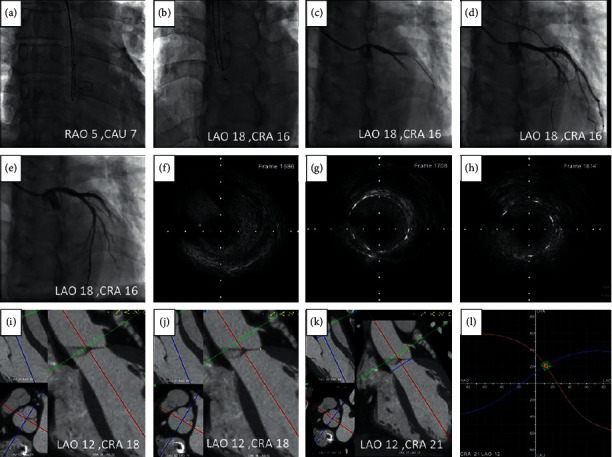
(a) Two 0.035-inch wires were advanced to the NCC and RCC using the anterior-posterior view; (b) the lowest points in the curve at the distal part of these two wires were superimposed at LAO18/CRA16; (c) LMCA angiography was performed at the angulation; (d) the stent was located at the angulation; (e) the image of LMCA after stent deployed; (f) LMCA ostial lesion was showed by intravascular ultrasound (OptiCrossTM HD; Boston Scientific) that was withdrawn at a pullback speed of one millimeter equal to 60 frames before stenting; (g, h) in the poststenting pullback, the LMCA ostium was at frame 1708 and the stent ostium was at frame 1814. So the stent protruded to the aorta for 1.77 mm ((1814–1708)/60); (i) the NCC and RCC overlapped at LAO12/CRA18 by FluoroCT; (j) the LMCA ostium faced to the NCC-RCC commissure in this patient (bottom left corner); (k) blue line was aortic valve plane and orange line was LMCA plane; (l) the two S-curves consisting of an unlimited number of pairs of C-arm angulations which were tangential to the aortic annulus and LMCA ostium crossed at LAO12/CRA21. CAU = caudal; CRA = cranial; LAO = left anterior oblique; RAO = right anterior oblique.

In all the 50 patients enrolled in our study, only one patient had a LMCA ostial lesion, and a stent was deployed on the LMCA guided by our approach. The procedure is shown in Figure 7. For this patient, the 3D angulation deviation was 7.6^o^, which was between LAO 18/Cranial 16 and LAO 12/Cranial 21 for optimal projection to visualize the LMCA ostium between the fluoroscopic and the 3D-CT reconstruction approaches.

#### 3.4.2. Example Two

**Figure 8:**
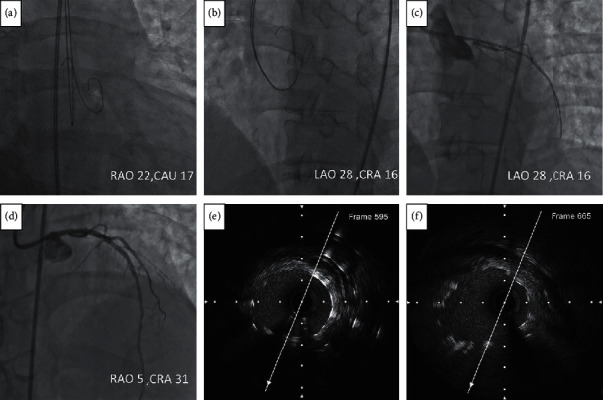
(a) Two 0.035-inch wires were advanced to the NCC and RCC using the RAO and CAU view; (b) the lowest points in the curve at the distal part of these two wires were superimposed at LAO 28/CRA 16; (c) the stent was located at the angulation; (d) the image of LMCA after stent deployed at RAO 5/CRA 31; (e) in the poststenting pullback with the speed of one millimeter equal to 60 frames, the LMCA ostium was at frame 595; (f) the stent ostium was at frame 665. So the stent protruded to the aorta for 1.17 mm ((665–595)/60). CAU = caudal; CRA = cranial; LAO = left anterior oblique; RAO = right anterior oblique.

The patient who had a LMCA ostial lesion was not enrolled in our study because he had no coronary CT scan before coronary angiography. The intervention for the LMCA ostial lesion was performed using the approach to generate the optimal projection to visualize the LMCA ostium guided by the intravascular ultrasound. The procedure was shown in Figure 8.

## 4. Discussion

In this study, the optimal projection to visualize the LMCA ostium was estimated with good accuracy using fluoroscopic images of wires in the NCC and RCC if the LMCA ostium faced the NCC-RCC commissure, which was the case in 71% of the patients studied.

The normal aorta valve is composed of three cusps, namely, the left coronary cusp (LCC), RCC, and NCC, which are symmetrically located in the aortic root [8]. The percentage of patients with only two coronary cusps is very low [9, 10]. In most patients the LMCA takes off from the middle part of the LCC [11]. The LMCA ostium usually faces the NCC-RCC commissure [12]. Fluoroscopically, the plane which crosses the LMCA ostium and the NCC-RCC commissure should be fixed when superimposing the nadirs of the NCC and RCC, which can be seen fluoroscopicly when the wires are advanced to the NCC and RCC. The angulation of the C-arm at this position will be the optimal projection to visualize the LMCA ostium.

For validation, the optimal projection to visualize the LMCA ostium was determined using 3D-CT reconstruction software. Using CT, Hell et al. showed an optimal angulation of LAO 23° ± 21°/Cranial 25° ± 23° (90% of patients had LAO/Cranial angulation, 3% LAO/Caudal, 4% RAO/Cranial, and 3% RAO/Caudal) for the LMCA ostium [4]. Also using CT, Kočka et al. reported that the average optimal projection to view the LMCA ostium was LAO 37^o^/Cranial 22° [5]. In all the abovementioned studies, the optimal projection to visualize LMCA ostium was provided as an average value, thereby only providing a tendency angle for exposing the LMCA ostium during coronary angiography for patients without a coronary CT scan before the angiography guided procedure.

## 5. Limitations

Quality images could be obtained in only 45 of 50 patients; occasionally, the wires could not be advanced into the NCC and RCC or remain stable in the RCC. Although hydrophilic wires might perform better, it is harder to control their posing. The method will not work if the three coronary cusps are not symmetrically distributed.

## 6. Conclusions

It is feasible to determine the optimal fluoroscopic projection to visualize the LMCA ostium by superimposing images of wires in the NCC and RCC when the LMCA ostium faces the NCC-RCC commissure.

## Data Availability

The clinical data used to support the findings of this study are available from the corresponding author upon request.

## Supplementary Materials

Supplementary Materials Comparisons of the noncoronary cusp (NCC) and right coronary cusp (RCC) overlapping and the optimal projection to visualize the left main coronary artery (LMCA) ostium between fluoroscopy and computed tomography were provided in three situations. The first was when the LMCA ostium faced the NCC-RCC commissure; the second was when the LMCA ostium faced the RCC; and the third was when the LMCA ostium faced the NCC.
