# Supplementary material for: Determination of Optimal Fluoroscopic Angulations for Left Main Coronary Artery Ostial Interventions: 3-Dimensional Computed Tomography Validation
Source: J Interv Cardiol. 2022 Mar 10;2022:2411824. doi: 10.1155/2022/2411824 (PMC8930230; doi:10.1155/2022/2411824)
Supplement: Supplementary Materials — Comparisons of the noncoronary cusp (NCC) and right coronary cusp (RCC) overlapping and the optimal projection to visualize the left main coronary artery (LMCA) ostium between fluoroscopy and computed tomography were provided in three situations. The first was when the LMCA ostium faced the NCC-RCC commissure; the second was when the LMCA ostium faced the RCC; and the third was when the LMCA ostium faced the NCC. [file 2411824.f1.docx]

Comparisons of the non-coronary cusp (NCC) and right coronary cusp (RCC) overlapping and the optimal projection to visualize the left main coronary artery (LMCA) ostium between fluoroscopy and computed tomography (CT) when the LMCA ostium faced the NCC-RCC commissure or when the LMCA ostium did not face the NCC-RCC commissure

1. When the LMCA ostium faced the NCC-RCC commissure
   1. Comparison of the NCC and RCC overlapping between Fluoroscopy and CT

Thirty-two in 45 LMCA ostia faced the NCC-RCC commissure. Mean fluoroscopy-derived angles of the overlapping NCC and RCC was 21.8±10.5 while the mean FluoroCT-derived angle of the overlapping NCC and RCC was 23.5±10.9 at left anterior oblique (LAO). Mean fluoroscopy-derived angles of the overlapping NCC and RCC was 20.1±9.0 while the mean FluoroCT-derived angle of the overlapping NCC and RCC was 21.4±7.5 at Cranial. Bland-Altman analysis showed a consistency between these two methods. Mean difference for the angle of the overlapping NCC and RCC at horizontal axes was -1.7 with a 95% limit of agreement between -4.27 and 0.96(p=0.21). Mean difference for the angle of the overlapping NCC and RCC at vertical axes was -1.3 with a 95% limit of agreement between -3.56 and 1.00(p=0.26) (Figure 1(a) and 1(b)).

- 1. Comparison of the optimal projection to visualize the LMCA ostium between Fluoroscopy and CT

Mean fluoroscopy-derived the optimal projection to visualize the LMCA ostium was 21.8±10.5 while the mean FluoroCT-derived angle of the overlapping NCC and RCC was 23.6±11.7 at LAO. Mean fluoroscopy-derived angles of the overlapping NCC and RCC was 20.1±9.0 while the mean FluoroCT-derived angle of the overlapping NCC and RCC was 21.7±8.0 at Cranial. Bland-Altman analysis showed a consistency between these two methods. Mean difference for the optimal projection to visualize the LMCA ostium at horizontal axes was -1.7 with a 95% limit of agreement between -4.45 and 1.01(p=0.21). Mean difference for the optimal projection to visualize the LMCA ostium at vertical axes was -1.6 with a 95% limit of agreement between -4.08 and 0.89(p=0.20) (Figure 1(c) and 1(d)).

1. When the LMCA ostium faced the RCC
   1. Comparison of the NCC and RCC overlapping between Fluoroscopy and CT

Nine in 45 LMCA ostia faced the RCC. Mean fluoroscopy-derived angles of the overlapping NCC and RCC was 23.4±11.1 while the mean FluoroCT-derived angle of the overlapping NCC and RCC was 25.3±9.0 at LAO. Mean fluoroscopy-derived angles of the overlapping NCC and RCC was 15.1±5.9 while the mean FluoroCT-derived angle of the overlapping NCC and RCC was 18.2±5.9 at Cranial. Bland-Altman analysis showed a consistency between these two methods. Mean difference for the angle of the overlapping NCC and RCC at horizontal axes was -1.9 with a 95% limit of agreement between -8.06 and 4.28(p=0.5). Mean difference for the angle of the overlapping NCC and RCC at vertical axes was -3.1 with a 95% limit of agreement between -8.13 and 1.91(p=0.19) (Figure 2(a) and 2(b)).

- 1. Comparison of the optimal projection to visualize the LMCA ostium between Fluoroscopy and CT

Mean fluoroscopy-derived the optimal projection to visualize the LMCA ostium was 23.4±11.1 while the mean FluoroCT-derived angle of the overlapping NCC and RCC was 38.7±14.4 at LAO. Mean fluoroscopy-derived angles of the overlapping NCC and RCC was 15.1±5.9 while the mean FluoroCT-derived angle of the overlapping NCC and RCC was 27.8±13.5 at Cranial. Bland-Altman analysis showed an inconsistent tendency in mean difference between these two methods for the optimal projection to visualize the LMCA ostium at horizontal axes, which was -15.2 with a 95% limit of agreement between -31.15 and 0.71(p=0.06), while an inconsistency in the mean difference, which was -23.6 with a 95% limit of agreement between -35.56 and -11.55(p=0.002) at vertical axes (Figure 2(c)and 2(d)).

1. When the LMCA ostium faced the NCC
   1. Comparison of the NCC and RCC overlapping between Fluoroscopy and CT

Four in 45 LMCA ostia faced the RCC. Mean fluoroscopy-derived angles of the overlapping NCC and RCC was 22.0±5.3 while the mean FluoroCT-derived angle of the overlapping NCC and RCC was 24.8±6.7 at LAO. Mean fluoroscopy-derived angles of the overlapping NCC and RCC was 27.8±9.6 while the mean FluoroCT-derived angle of the overlapping NCC and RCC was 28.5±7.7 at Cranial. Bland-Altman analysis showed a consistency between these two methods. Mean difference for the angle of the overlapping NCC and RCC at horizontal axes was -2.7 with a 95% limit of agreement between -11.11 and 5.60(p=0.37). Mean difference for the angle of the overlapping NCC and RCC at vertical axes was -0.8 with a 95% limit of agreement between -8.69 and 7.19(p=0.78) (Figure 3(a) and 3(b)).

- 1. Comparison of the optimal projection to visualize the LMCA ostium between Fluoroscopy and CT

Mean fluoroscopy-derived the optimal projection to visualize the LMCA ostium was 22.0±5.3 while the mean FluoroCT-derived angle of the overlapping NCC and RCC was 10.3±11.2 at LAO. Mean fluoroscopy-derived angles of the overlapping NCC and RCC was 27.8±9.6 while the mean FluoroCT-derived angle of the overlapping NCC and RCC was 12.5±4.4 at Cranial. Bland-Altman analysis showed a consistency in mean difference between these two methods for the optimal projection to visualize the LMCA ostium at horizontal axes, which was 11.8 with a 95% limit of agreement between -7.88 and 31.38(p=0.15), while an inconsistency in the mean difference for the optimal projection to visualize the LMCA ostium at vertical axes，which was 15.3 with a 95% limit of agreement between 5.32 and 25.18(p=0.02) (Figure 3(c) and 3(d)).

**Figure 1.** Comparison of the NCC and RCC overlapping between coordinates generated using each method on (a) horizontal and (b) vertical axes and comparison of the optimal projection to visualize the LMCA ostium between coordinates generated by each method on (c) horizontal and (d) vertical axes when the LMCA ostium faced the NCC-RCC commissure.

**Figure 2.** Comparison of the NCC and RCC overlapping between coordinates generated using each method on (a) horizontal and (b) vertical axes and comparison of the optimal projection to visualize the LMCA ostium between coordinates generated by each method on (c) horizontal and (d) vertical axes when the LMCA ostium faced the RCC.

**Figure 3.** Comparison of the NCC and RCC overlapping between coordinates generated using each method on (a) horizontal and (b) vertical axes and comparison of the optimal projection to visualize the LMCA ostium between coordinates generated by each method on (c) horizontal and (d) vertical axes when the LMCA ostium faced the NCC.
